# Different Principles of ADP-Ribose-Mediated Activation and Opposite Roles of the NUDT9 Homology Domain in the TRPM2 Orthologs of Man and Sea Anemone

**DOI:** 10.3389/fphys.2017.00879

**Published:** 2017-10-31

**Authors:** Frank Kühn, Cornelia Kühn, Andreas Lückhoff

**Affiliations:** Medical Faculty, Institute of Physiology, RWTH Aachen University, Aachen, Germany

**Keywords:** *Nematostella vectensis*, ADP-ribose, calcium, oxidative stress, 2-APB

## Abstract

A decisive element in the human cation channel TRPM2 is a region in its cytosolic C-terminus named NUDT9H because of its homology to the NUDT9 enzyme, a pyrophosphatase degrading ADP-ribose (ADPR). In *h*TRPM2, however, the NUDT9H domain has lost its enzymatic activity but serves as a binding domain for ADPR. As consequence of binding, gating of the channel is initiated. Since ADPR is produced after oxidative DNA damage, *h*TRPM2 mediates Ca^2+^ influx in response to oxidative stress which may lead to cell death. In the genome of the sea anemone *Nematostella vectensis (nv)*, a preferred model organism for the evolution of key bilaterian features, a TRPM2 ortholog has been identified that contains a NUDT9H domain as well. Heterologous expression of *nv*TRPM2 in HEK-293 cells reveals a cation channel with many close similarities to the human counterpart. Most notably, *nv*TRPM2 is activated by ADPR, and Ca^2+^ is a co-agonist. However, the intramolecular mechanisms of ADPR gating as well as the role of NUDT9H are strikingly different in the two species. Whereas already subtle changes of NUDT9H abolish ADPR gating in *h*TRPM2, the region can be completely removed from *nv*TRPM2 without loss of responses to ADPR. An alternative ADPR binding site seems to be present but has not yet been characterized. The ADP-ribose pyrophosphatase (ADPRase) function of *nv*NUDT9H has been preserved but can be abolished by numerous genetic manipulations. All these manipulations create channels that are sensitive to hydrogen peroxide which fails to induce channel activity in wild-type *nv*TRPM2. Therefore, the function of NUDT9H in *nv*TRPM2 is the degradation of ADPR, thereby reducing agonist concentration in the presence of oxidative stress. Thus, the two TRPM2 orthologs have evolved divergently but nevertheless gained analogous functional properties, i.e., gating by ADPR with Ca^2+^ as co-factor. Opposite roles are played by the respective NUDT9H domains, either binding of ADPR and mediating channel activity, or controlling the availability of ADPR at the binding site located in a different domain.

## Human TRPM2: history and hallmarks of an exceptional cation channel

The scientific community was very much excited when in 2001, Perraud et al. reported that the human Ca^2+^-permeable cation channel LTRPC2 was activated by intracellular ADP-ribose. Two characteristics of this channel, renamed in the meantime to TRPM2, were particularly fascinating. The first one is its activation by ADPR, a metabolite which had not been on the list of potential stimuli of ion channels at this time, although it was known to induce the fertilization current in oocytes of the sea squirt *Ciona intestinalis* (Wilding et al., 1998). From then on it was quickly realized that ADPR, produced in response to oxidative stress and as consequence of DNA damage, mediates Ca^2+^ influx through TRPM2 channels which may eventually lead to apoptosis or other forms of cell death. Second, TRPM2 contains a homology region within the cytosolic C-terminus that strongly resembles the human NUDT9 pyrophosphatase as well as homologous bacterial enzymes of the NUDIX-family (Bessman et al., 1996; Perraud et al., 2003). Hence, TRPM2 might be considered a “chanzyme,” a channel protein that additionally displays enzymatic activity intimately linked to channel function.

It has been well established that ADPR is the principal activator of TRPM2. Few related substances have been reported to share its agonistic properties (Grubisha et al., 2006; Tóth et al., 2014, 2015; Fliegert et al., 2017). However, a major role as an essential co-factor is played by Ca^2+^ (McHugh et al., 2003; Starkus et al., 2007; Csanády and Töröcsik, 2009). For an effective stimulation by ADPR, Ca^2+^ must be presented either on the extracellular or the intracellular side of the plasma membrane and its action is likely to take place within the pore region (as discussed later). In particular, intracellular Ca^2+^ strongly modulates the sensitivity of TRPM2 to ADPR, to an extent that in neutrophil granulocytes activation of TRPM2 occurs without an apparent increase in the intracellular concentration of ADPR as soon as intracellular Ca^2+^ is elevated (Heiner et al., 2006). Since basal levels of ADPR are sufficient to enable Ca^2+^-directed TRPM2 gating, ADPR renders TRPM2 a Ca^2+^-activated cation channel that is indispensible for some but not all responses of neutrophils during antibacterial defense. Especially chemotaxis seems to be critically dependent on the preceding stimulation of TRPM2 and is significantly impaired in TRPM2 knock-out mice (Sumoza-Toledo et al., 2011).

Neutrophil granulocytes are among the few cells that are not equipped with poly(ADP-ribose)-Polymerases (PARPs; Sanghavi et al., 1998). Along with poly(ADP-ribose) glycohydrolases (PARGs), these are key enzymes involved in the formation of ADPR after oxidative damage to the DNA (e.g., reviewed by Yamamoto and Shimizu, 2016). Therefore, in many other cell types including neurons, ADPR-induced Ca^2+^ influx through TRPM2 is a decisive element in the process that terminates in apoptosis after initiation by oxidative stress. Experimentally, oxidative stress is frequently induced by extracellular application of hydrogen peroxide (H_2_O_2_) to the cells. Indeed, in cell models with overexpression of TRPM2, H_2_O_2_ is a well-established stimulus of Ca^2+^ influx (Hara et al., 2002; Wehage et al., 2002). Another extracellularly applicable stimulus of TRPM2 (as opposed to the strictly intracellular application of ADPR) is N-Methyl-N′-nitro-N-nitrosoguanidine (MNNG) that, like H_2_O_2_, is an activator of PARP-1 (Buelow et al., 2008; Chiu et al., 2011). It is believed that the action of H_2_O_2_ is an indirect one, depending on the intracellular accumulation of ADPR (Perraud et al., 2005). Consequently, current induction in response to H_2_O_2_ takes some time, in contrast to the fast onset after stimulation of TRPM2 with high concentrations of intracellular ADPR during patch-clamp experiments. The co-operation of ADPR and Ca^2+^, along with the positive feed-back constituted by Ca^2+^ entry through already activated TRPM2 channels (McHugh et al., 2003; Heiner et al., 2006; Csanády and Töröcsik, 2009; Tóth and Csanády, 2010), explains why any effect of H_2_O_2_ is strongly dependent on Ca^2+^, even more strictly than under experimental conditions when ADPR in excess is directly applied to the channel. Therefore, an elevated intracellular Ca^2+^ concentration of 1 μM is routinely used in our lab in patch-clamp experiments when TRPM2 or TRPM2 variants are tested for sensitivity toward H_2_O_2_.

Mammalian TRPM2 channels are moreover sensitive to temperature (Togashi et al., 2006; Kashio et al., 2012) and are apparently involved in temperature sensing (Song et al., 2016; Tan and McNaughton, 2016), but this is beyond the scope of this review, as is its role as a channel in membranes of intracellular organelles (Lange et al., 2009).

TRPM2 so far is the only ion channel that is directly activated by ADPR. This should not be confused with other regulatory functions of ADPR, notably ADP ribosylation, which takes place, e.g., in the purinergic P2X7 receptor and leads to channel activation (Adriouch et al., 2008). Among the four known “chanzymes,” the most prominent member is still the cystic fibrosis transmembrane conductance regulator (CFTR) channel (Ramjeesingh et al., 1999), whereas the other three ones all belong to the melastatin-subfamily of TRP channels (Perraud et al., 2001; Runnels et al., 2001; Schlingmann et al., 2002). Until recently, it was believed that the catalytic activity of the respective enzyme domain contributes decisively to their gating process. However, now it seems clear that for TRPM6 and TRPM7, the enzyme domain is not really essential for gating but rather performs a regulatory function (Matsushita et al., 2005; Thébault et al., 2008; Cai et al., 2017). In the case of CFTR and also of human TRPM2, multiple lines of evidence suggest that not catalysis but binding of the substrate represents the critical step for channel activation (Tóth et al., 2014; Mihályi et al., 2016).

**Figure 1:**
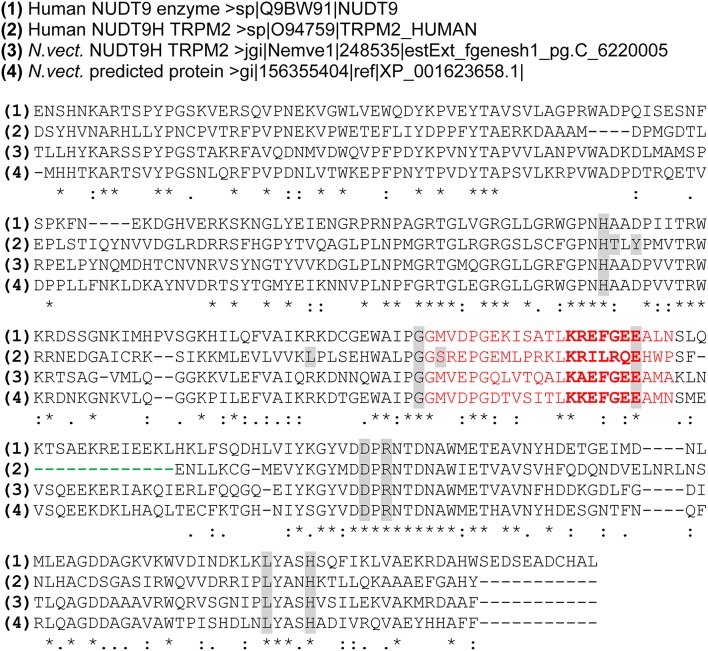
Multiple Sequence alignment of the NUDT9-related channel domains of *h*TRPM2 and *nv*TRPM2 and the NUDT9 enzymes of man and sea anemone. Alignment was performed (using the tool at www.uniprot.org/align/) with amino acid sequences of the human NUDT9 enzyme (aa 59–350) the NUDT9 homology (NUDT9H) domains of human TRPM2 (aa 1236–1503) and sea anemone TRPM2 (aa 1,271–1,551) as well as of the putative NUDT9 enzyme of the sea anemone (aa 1–281). The NUDIX sequence motif containing the catalytic active site (bold) is given in red. The functionally important deletion downstream of the NUDIX box which is exclusively present in *h*TRPM2 is indicated with a green dashed line. Individual residues found to be directly involved in the binding of ADP-ribose in *h*TRPM2 (Yu et al., 2017) are in gray. Most of these residues are conserved in the corresponding region of *nv*TRPM2 and the NUDT9 enzymes. Symbols (^*^, :, .) denote the degree of conservation observed in each column as specified on the website indicated above.

The principal structure of the NUDT9 homology domain of TRPM2 which is very similar to the almost identical NUDT9 enzymes of man and sea anemone is outlined in Figure 1. The more C-terminally localized catalytic center is formed by a strongly conserved amino acid sequence, the so-called NUDIX-box (Bessman et al., 1996). It has been experimentally demonstrated that the two successive amino acid residues glutamate-phenylalanine of this region are especially important for the activity of the human enzyme (Perraud et al., 2003). If these residues are mutated to isoleucine-leucine, the activity is reduced to about 1% (Shen et al., 2003). Exactly this substitution is present in the NUDT9H domain of human TRPM2 which strongly suggests that the enzymatic activity has been largely abolished while NUDT9 has undergone the adaption to a channel domain of TRPM2. Importantly, the reciprocal mutation of the critical sequence of TRPM2 back to that of the NUDT9 enzyme abolishes any channel function (Kühn and Lückhoff, 2004; Perraud et al., 2005; Du et al., 2009) suggesting that ADPR hydrolysis and channel function are incompatible with each other. Moreover, channel activation can be readily achieved with the non-hydrolyzable ADPR analog alpha, beta-methylene ADP-ribose (AMPCPR) (Tóth et al., 2014). Taken together, there is ample and strong evidence for the notion that catalytic activity is not necessary and even detrimental for the activation of TRPM2.

The currently favored view is that the NUDT9H region of TRPM2 ensures the specific binding of the channel agonist ADPR. Several studies have clearly demonstrated that already subtle changes within the structure of the NUDT9H domain may lead to a complete loss of channel function (e.g., Kühn and Lückhoff, 2004; Perraud et al., 2005). Obviously, binding and subsequent channel gating have very restricted structural requirements that can easily be disturbed.

## Studies on orthologous channels from distantly related species to get mechanistic insight

While it is generally accepted and well documented that gating of TRPM2 requires binding of ADPR to the NUDT9H domain, the subsequent steps that ultimately lead to pore opening are far from being understood. Methodological approaches that would provide a straightforward interpretation are not obvious. In this situation, a strategy may be helpful that has been successfully employed for several other ion channels: the structural and functional comparison of species variants. Prominent examples are the capsaicin receptor TRPV1 (Jordt and Julius, 2002), the menthol receptor TRPM8 (Chuang et al., 2004) and the chemoreceptor TRPA1 (Laursen et al., 2015).

In this review, we will summarize the findings and perspectives gained from studies on the TRPM2 ortholog of the sea anemone *Nematostella vectensis*.

Of course, the question arises why choosing the sea anemone as species variant. There are several good reasons for this choice. First, the evolution especially of the TRPM channel subfamily seems to have taken a very interesting course. In basal metazoans and even in unicellular protists, there is only one representative of the TRPM subfamily and this is clearly classified as TRPM2-like (Mederos y Schnitzler et al., 2008). This and other indications allow the conclusion that in the beginning of the metazoan evolution, a TRPM2-like channel stepped on stage which probably represents the evolutionary ancestor of all modern TRPM channels. This scenario implicates that the archetypal TRPM channel should have had structural and functional features that are at least partially present in all modern TRPM subtypes, including TRPM2. *Nematostella vectensis* today represents a preferred model organism for the study of the evolution of some archetypal metazoan blueprints such as the immune system and the nervous system. Especially for comparative studies on TRPM2, it is noteworthy that the natural habitats of *Nematostella vectensis* are salt marshes along the coasts of the northern Atlantic Ocean. Here, animals are commonly exposed to UV radiation and diverse chemicals, all of which can exert oxidative stress (Tarrant et al., 2014). Because the mammalian TRPM2 ortholog represents a central player in the process of oxidative-stress mediated apoptosis, the suitability of *Nematostella vectensis* as a simplistic model appears evident.

The comparative studies on *h*TRPM2 and *nv*TRPM2 reveal that both of these far distantly related channel orthologs are activated by ADPR. However, and unexpectedly, this is achieved by vastly different mechanisms and parts of the channel protein.

## Topical and detailed structure of *nv*TRPM2

The genome of the starlet sea anemone *Nematostella vectensis* was sequenced and assembled by whole genome shotgun by Putnam et al. (2007). A search of Nudix-linked TRPM proteins in genomic sequence databases by Mederos y Schnitzler et al. (2008) revealed that they are invariably present in chordates, molluscs, echinodermates and also in basal metazoans like cnidarians and even in unicellular protists. As the complete expressed sequence tag (start codon to stop codon open reading frame) of the sea anemone TRPM2-like channel was published in the joint genome institute database (jgi.Nemve1.248535|estExt_fgenesh1_pg.C_6220005), it was possible to make this gene available for functional expression in heterologous expression systems by commercial gene synthesis (Kühn et al., 2015).

The sea anemone TRPM2 open reading frame (ORF) contains 1551 amino acid residues (aa) and on closer inspection represents the only full-length TRPM gene product of *Nematostella vectensis* (Mederos y Schnitzler et al., 2008; Peng et al., 2015). The sea anemone TRPM2 open reading frame displays a total sequence identity of 31% to the corresponding sequence of human TRPM2 (1503 aa). The similarity is greatest in the N-terminal region upstream of the putative transmembrane segments (36% identity) and in the NUDT9H domain (39% identity), whereas the regions containing the transmembrane segments (25% identity) and the connecting linker to the NUDT9H domain (27% identity) are less conserved. Furthermore, the NUDT9H domain of *nv*TRPM2 (aa 1271–1,551) shows 49% sequence identity to the corresponding sequence of the *h*NUDT9 enzyme (aa 59–350) which is notably higher than between the *h*NUDT9 enzyme and NUDT9H (aa 1,236–1,503) of *h*TRPM2 (34%; Kühn et al., 2015; see also Figure 1). Compared to the *h*NUDT9-enzyme, in both *nv*TRPM2 and *h*TRPM2, the putative ADPR binding domain of the NUDT9H domain is well conserved, including the critical residue N1326 of *h*TRPM2 (Kühn and Lückhoff, 2004; Kühn et al., 2016). However, the active site of the *h*NUDT9 enzyme containing the NUDIX box signature GX_5_EX_7_REUXEEXGU (Bessman et al., 1996) is slightly different in NUDT9H of *nv*TRPM2 and markedly different in NUDT9H of *h*TRPM2 (Figure 1). This fact strongly suggests that the NUDT9H domain of *nv*TRPM2, in contrast to the *h*TRPM2 counterpart, is very likely to have a largely intact catalytic function.

A short amino acid motif within the proximal part of the predicted pore loop contributes significantly to the Ca^2+^ permeation of enzyme-linked TRPM channels (Mederos y Schnitzler et al., 2008). In the non-selective group, among them *h*TRPM2, this motif consists of the amino acid triplet glutamine-isoleucine-proline (QIP), whereas in the more Ca^2+^-selective members, as for example TRPM7, this motif is changed to glutamate-valine-tryptophane (EVY). In general, the TRPM2-like channels of diverse organisms ranging from choanoflagellates to primitive chordates and also *nv*TRPM2 contain the motif glutamate-leucine-phenylalanine (ELF) which indicates the signature of a more Ca^2+^-permeable channel (Mederos y Schnitzler et al., 2008).

As a striking difference to the primary structure of *h*TRPM2, the *nv*TRPM2 channel exhibits a much longer S1-S2 linker region with numerous glutamate and lysine residues. Notably, this region shows significant similarity to the corresponding region of the *h*TRPM3 channel which strengthens the hypothesis that a TRPM2-like channel represents a common ancestor of the contemporary TRPM-subfamily (Mederos y Schnitzler et al., 2008; Kühn et al., 2015).

## Functional expression of *nv*TRPM2 in human cells reveals cation currents induced by ADPR and by Ca^2+^

From the overall high topological similarity between *nv*TRPM2 and mammalian TRPs, we were confident in the beginning of our studies that the sea anemone ortholog could be functionally overexpressed with standard methods in mammalian cells, although until then only few examples existed where a successful heterologous expression of such far distantly related ion channels had been achieved and this was in oocytes of *Xenopus laevis* (e.g., Jegla et al., 2012; Assmann et al., 2014; Baker et al., 2015). The standard procedure of commercially available gene synthesis was used and the codon usage was adapted to the human expression system (Ikemura, 1985). This manipulation is frequently a prerequisite for the successful heterologous expression of proteins from distantly related species. The successful expression of all TRPM2 channels (human or *Nematostella* orthologs) in HEK-293 cells was verified by cell surface biotinylation assay and Western-blot-analysis with variants containing hemagglutinin (HA) tags downstream from the respective open reading frame (Kühn et al., 2016). This procedure was chosen to minimize the danger of artifacts due to species-specific antibodies. Wild-type and mutant *nv*TRPM2 channels were expressed in the plasma membrane with no obvious difference to the human ortholog.

For functional analysis, mostly the variants without HA tags were studied using the standard whole-cell patch-clamp technique. The non-electrophysiologists among the readers should understand that with this technique, the cytosol of the cells is replaced with the solution in the pipette within seconds by diffusion. Thus, the intracellular concentrations of the standard stimulus, ADPR, as well as the intracellular concentration of Ca^2+^ is completely controlled by the composition of the pipette fluid. For some selected *nv*TRPM2 variants, the biophysical properties were explored with single-channel analysis in inside-out patches (Kühn et al., 2016).

The electrophysiological studies demonstrate that *nv*TRPM2 is expressed in HEK-293 cells as fully functional cation channel activated by ADPR and by its co-agonist Ca^2+^. Thus, the principal activators of *h*TRPM2 are effective in the ortholog of a distantly related species as well. In addition to many common features of ADPR-induced currents, however, there were several properties unique for *nv*TRPM2. These include in the first line the concentration-effect-relation as well as the on and off kinetics.

**Figure 2:**
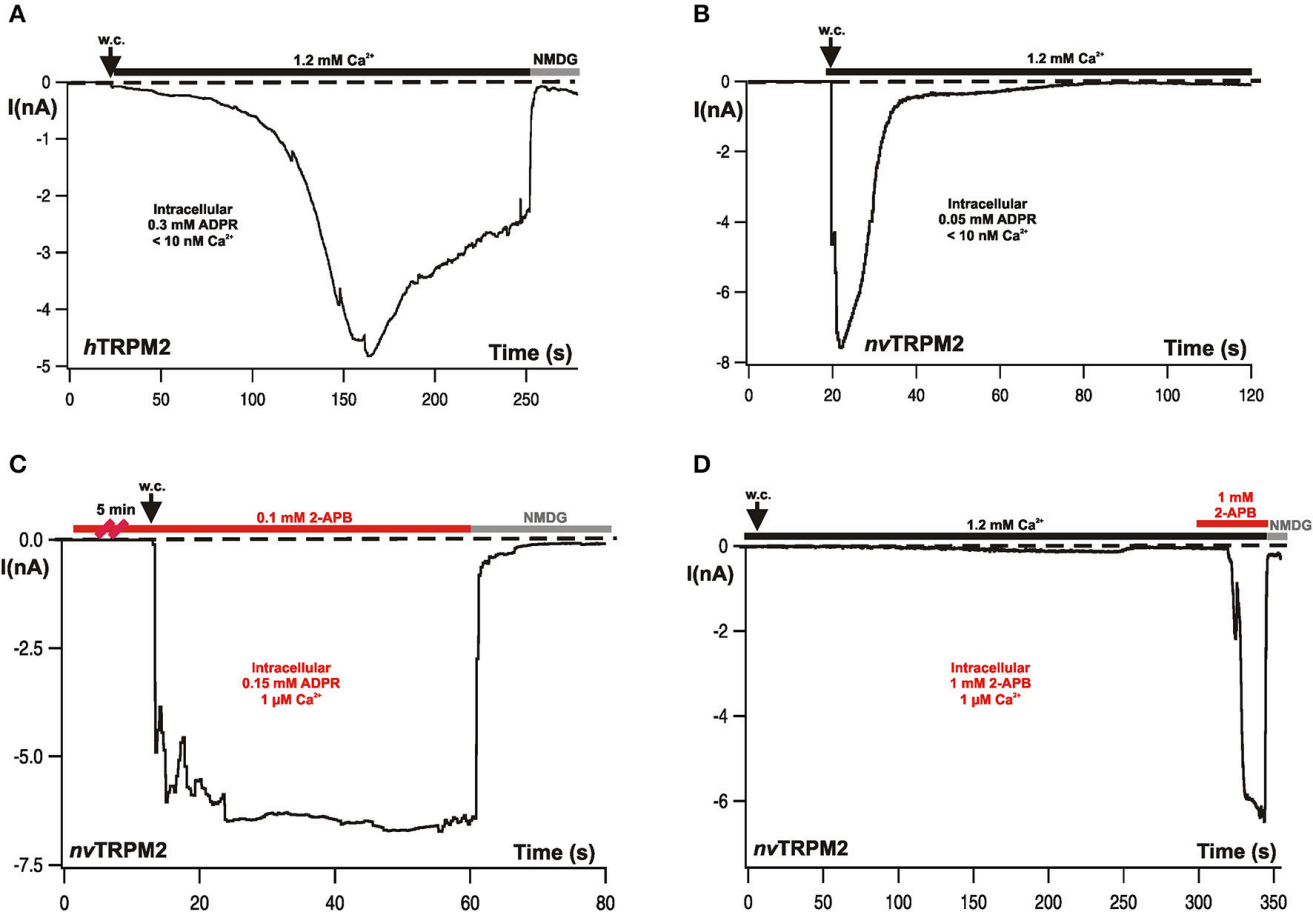
Typical whole-cell patch-clamp recordings from *h*TRPM2 and from *nv*TRPM2 channels in HEK-293 cells. **(A)** Characteristic currents of *h*TRPM2 during stimulation with ADPR infused through the patch-pipette. Intracellular (pipette) concentrations of ADPR and Ca^2+^ are indicated. Both activation and inactivation kinetics are comparatively slow. At the end of the experiment, the currents were fully blocked by a substitution of extracellular monovalent cations with NMDG. **(B)** Currents of *nv*TRPM2 in response to ADPR showing the characteristic fast kinetics of activation and inactivation. **(C)** Similar experimental conditions as shown before but the cells had been pre-incubated for at least 5 min in bath solution containing 2-APB (0.1 mM). The rapid inactivation is completely suppressed. **(D)** No stimulation of *nv*TRPM2 with 2-APB (1 mM) instead of ADPR in the pipette solution. Only when applied from the extracellular side of the plasma membrane high concentrations of 2-APB (≥1 mM) induces a strong and fast current activation after a characteristic delay, while inactivation does not occur. The standard Ca^2+^ concentration in the bath solution for the measurements is 1.2 mM Ca^2+^. Figures are slightly modified from Kühn et al. (2015, 2016, 2017).

In human TRPM2, stimulation with ADPR results in a current that reaches its maximum within several tens of seconds. A run-down takes place over several minutes and is usually incomplete within the time frame of the experiments (Figure 2A). The amplitudes and kinetics depend significantly on Ca^2+^ which must be present on at least one side of the plasma membrane for the induction of any current (Perraud et al., 2001; McHugh et al., 2003; Starkus et al., 2007; Csanády and Töröcsik, 2009; Kühn et al., 2010). The elevation of the Ca^2+^ concentration either on the extracellular or the intracellular side fail to stimulate TRPM2 channels in the absence of ADPR. On the other hand, removal of Ca^2+^ from the extracellular side promptly abolishes ADPR-induced currents when Ca^2+^ is absent in the cytosol. These two findings establish the role of ADPR and Ca^2+^ as essential co-agonists. In the presence of 1 μM Ca^2+^ in the cytosol, half-maximal current amplitudes are reached with ADPR concentrations of about 100 μM. The ADPR concentration needs to be increased to 500 μM when Ca^2+^ is removed from the pipette fluid.

In characteristic distinction to *h*TRPM2, the *Nematostella* ortholog *nv*TRPM2 displays much faster developing currents of large amplitude after stimulation with ADPR; however, the currents return to baseline within a few seconds (Figure 2B). These responses are induced by already moderate concentrations of ADPR (25–50 μM) in the absence of intracellular Ca^2+^ (≤10 nM); ADPR concentrations as low as 10 μM were sufficient with 1 μM Ca^2+^. No currents were observed when Ca^2+^ was missing on both sides of the plasma membrane (Kühn et al., 2015). Therefore, Ca^2+^ is an essential co-factor as in *h*TRPM2. However, one order of magnitude less ADPR is required for *nv*TRPM2 than for *h*TRPM2.

With respect to many biophysical properties, *nv*TRPM2 and *h*TRPM2 appear closely similar. Single channel open times are extremely long in inside-out patches with ADPR on the cytosolic side of the plasma membrane, frequently reaching several hundreds of milliseconds. Likewise, there is almost no discrimination between monovalent and divalent cations, as shown by the reversal potential close to 0 mV in asymmetric solutions (Kühn et al., 2015).

The on-kinetics, which is markedly accelerated by Ca^2+^ in the case of *h*TRPM2, was not modified in *nv*TRPM2 by increasing the concentrations of ADPR and Ca^2+^. This may have not been expected anyway because they are extremely fast for a ligand-gated channel already at standard conditions. Interestingly, the on-kinetics remained fast when the pore signature glutamate-leucine-phenylalanine (ELF) of *nv*TRPM2 was changed to glutamine-leucine-proline (QLP) which is characteristic for TRP channels with little Ca^2+^ permeability (Mederos y Schnitzler et al., 2008). It appears that although Ca^2+^ in the pore is essential, it is not required in high amounts or concentrations; a graded modulation of ADPR-induced currents by intracellular Ca^2+^ cannot be demonstrated experimentally.

Beyond the effects of Ca^2+^ on the on-kinetics of ADPR-dependent currents, Ca^2+^ has a strong impact on the off-kinetics. When extracellular Ca^2+^ is removed, ADPR induces currents that are sustained over extended periods of time. Already this finding suggests that Ca^2+^ exerts an action on the pore to induce a rapid current decline of the current.

In any case, Ca^2+^ entry profoundly affects the kinetics of ADPR-induced currents, whereas intracellular Ca^2+^ facilitates the principal activation in most experimental conditions.

Further evidence for this interpretation is discussed later in context of the effects of 2-APB. We therefore propose that the current decline should be referred to as inactivation because it relates to a pore-dependent mechanism. The term desensitization should, in our opinion, not be used because it may be understood to describe a process that affects binding of ADPR for which no experimental indication exists. Unfortunately, it is not easily possible to remove the stimulus ADPR during one experiment and repeat its application several times. In inside-out patches, this would be feasible; however, for some reasons that are not understood, single channel activity in response to ADPR persists much longer than whole-cell currents.

We have not performed a determination of the Ca^2+^ permeability deduced from reversal potentials in non-physiological high Ca^2+^ concentrations because this approach is unlikely to yield a true estimation of the contribution of Ca^2+^ to the total current under physiological ion conditions (Dzeja et al., 1999). However, studies on the QLP variant demonstrate that indeed Ca^2+^ access to the pore is improved by the ELF motif which, interestingly, preferentially concerns permission of activation by intracellular Ca^2+^, in co-operation with ADPR.

Taken together, *nv*TRPM is rapidly activated by ADPR and Ca^2+^ as co-agonists, with considerably higher sensitivity and faster kinetics than *h*TRPM2. A fast inactivation takes place through the action of Ca^2+^ entering the pore.

## 2-APB as a Ca^2+^-dependent gating modifier of TRPM2 channels

A general problem in the investigation of TRP channels, especially of the TRPM subfamily, is the lack of specific inhibitors. 2-Aminoethyl-diphenylborinate (2-APB) is one of the better candidates since its effects on TRPM channels are at least rapidly and completely reversible. On the other hand, the compound is by no means channel-specific and its effect can be inhibitory as well as activating. This depends on its concentration and the channel type. Even on one particular channel, it may exert both these opposite effects in a concentration dependent manner (e.g., Li et al., 2006; Jansen et al., 2016). Likewise, the human TRPM2 ortholog was exclusively inhibited already by moderate concentrations of 2-APB (0.1 mM), whereas the *nv*TRPM2 ortholog shows different and complex responses in the presence of 2-APB. In no case, an inhibition was observed; 2-APB left the activation by ADPR completely intact. However, the fast inactivation that is a characteristic feature of *nv*TRPM2 is completely abolished by the compound (Figure 2C) such that sustained currents are induced by ADPR in the presence of 2-APB (0.1–0.5 mM). At higher concentrations (1 mM), 2-APB activated large currents by itself (Figure 2D); again, these currents did not inactivate over time (Kühn et al., 2017).

Both these effects were strictly dependent on an extracellular application and were completely absent when 2-APB was present only in the pipette (intracellular) solution (Figure 2D; as also demonstrated for *h*TRPM2; Togashi et al., 2008; Kühn et al., 2017). This finding suggests that 2-APB acts on the channel pore; this view is supported by experiments on *nv*TRPM2 variants in which genetic manipulations have been performed in the pore region and which show altered responses to 2-APB, in comparison with wild-type *nv*TRPM2 (Kühn et al., 2017). We are convinced that it is a safe assumption that 2-APB indeed is a modifier of the pore properties and that it can therefore be used as a tool to explore these properties further, in particular with respect to Ca^2+^-mediated effects on the pore. The potential of 2-APB in this respect has not yet been fully exploited but already the initial results reveal surprising insight, as well as they give rise to further questions and to hypotheses that should be tested in the near future.

There are several key findings for 2-APB on *nv*TRPM2 that in combination result in a straightforward interpretation of its modes of action, although still some detailed questions remain open.

First, these are the peculiar on and off kinetics of 2-APB when used as a channel stimulus, i.e., in high concentrations. There is a lag time of several tens of seconds before any effect can be observed but afterwards, the development of currents occurs very rapidly within seconds. Wash-out of 2-APB, on the other hand, leads to an immediate cessation of the currents, much faster than their onset (Kühn et al., 2017). Thus, access to the pore is restricted as long as the channels are in a closed state but becomes fast as soon they are opened by 2-APB. As a result, an almost all-or-nothing kind of response to 2-APB is observed with an extremely steep concentration-response relation. While there is no apparent activation by 0.5 mM 2-APB, a full activation takes place if that concentration is doubled. Removal of 2-APB then lets the stimulus quickly leave the pore and the currents recede (Kühn et al., 2017).

The second key finding is the strict requirement on Ca^2+^ for the channel stimulation by 2-APB. Ca^2+^ must be present on both sides of the plasma membrane, in contrast to experiments with ADPR as stimulus of *nv*TRPM2 when either extracellular or intracellular Ca^2+^ was sufficient. It is tempting to speculate but not yet proven that this relates to multiple binding sites for Ca^2+^, as has been proposed for the pore of *h*TRPM2 (Csanády and Töröcsik, 2009). However, some clarification is gained by experiments with the QLP-variant of *nv*TRPM2. This mutation is supposed to change the pore signature to that of a less Ca^2+^ selective channel (Mederos y Schnitzler et al., 2008).

Indeed, this mutation seems to impede the access of Ca^2+^ to the pore, and not only from the extracellular but from the intracellular side as well. Removal of extracellular Ca^2+^, which has no dramatic effect on the stimulation of wild-type *nv*TRPM2, abolishes ADPR responses of *nv*TRPM2-QLP completely when the standard intracellular solution is used (with a Ca^2+^ concentration of 1 μM). Currents can be restored when intracellular Ca^2+^ is increased to 100 μM (a certainly non-physiological concentration). Likewise, the QLP variant was not stimulated by 2-APB when the extracellular Ca^2+^ concentration was normal. But with 10 mM Ca^2+^, again non-physiologically high, currents reappeared. The inactivation of ADPR-induced currents in the QLP variant was normal, suggesting that not so much Ca^2+^ is required for the inactivation as for the co-agonism with 2-APB. On the other hand, as co-agonist with ADPR, intracellular Ca^2+^ is more effective than extracellular one. These findings again may point to multiple Ca^2+^ binding sites within the pore with different functions, as more extensively discussed on *h*TRPM2 (Csanády and Töröcsik, 2009; Tóth and Csanády, 2012).

It should be kept in mind in this context that Ca^2+^ not only accesses the pore but that it permeates it. The latter, however, takes place only after opening of the channel. Access, on the other hand, is decisive prior to the channel's full activation and may occur in its closed state or when only few channel openings happen that do not produce a noticeable current but allow Ca^2+^ to reach its target within the pore. Again, Ca^2+^ mediates a self-enhancing process as a co-agonist for ADPR and for 2-APB because it leads to pore opening and at the same time its access is favored by pore opening. Moreover, whenever differences are observed between extracellular and intracellular Ca^2+^, it is difficult to decide whether these reflects steric reasons within the pore's architecture or merely a matter of the required Ca^2+^ concentration because the intracellular Ca^2+^ is low and cannot reasonably be increased too much.

The notion that Ca^2+^ within the pore is a prerequisite for channel activation not only by ADPR but by 2-APB as well is elegantly underlined by experiments on the QLP variant where a low concentration of ADPR is present in the pipette. A relatively small current is induced and inactivation takes place. Then, addition of 2-APB evokes currents with two remarkable properties. They are larger in amplitude than the previous ADPR-dependent ones, and they occur with a shorter delay than typical for 2-APB effects in the absence of ADPR. Our interpretation is that some Ca^2+^ has remained at the putative activating site in the pore and that the positive feedback of 2-APB and Ca^2+^ can now progress earlier.

An extremely interesting process in *nv*TRPM2 is the fast current inactivation which discriminates it from its human ortholog. In *h*TRPM2, the current decline after stimulation with ADPR is remarkably slow, such that the activation is frequently perceived as permanent. However, also in *h*TRPM2, inactivation may be important (Starkus et al., 2007), although at a different timescale. In *nv*TRPM2, inactivation takes place within fractions of a minute. As molecular mechanisms for this phenomenon, the experiments with 2-APB and on the pore mutant QLP provide strong evidence that inactivation represents processes within the pore and should therefore, as noticed before, referred to as inactivation, rather than desensitization. It is also clear that it is extracellular Ca^2+^ passing through the pore that mediates inactivation. Less clear is how this is prevented by 2-APB. The compound could interfere with the binding of Ca^2+^ to a (yet undefined) site specific for inactivation. Alternatively, it may be hypothesized that some Ca^2+^-induced pore collapse takes place as basis for inactivation. Such a mechanism has been proposed for *h*TRPM2 (Tóth and Csanády, 2012). As soon as 2-APB is present in the pore, collapse may be prevented without direct interference with Ca^2+^. In any case, the effects of 2-APB are immediately reversible after wash-out.

As a side-note with the potential of an experimental pitfall, we would like to add that 2-APB may interfere with the large cation NMDG in a manner that is difficult to interpret biologically but may lead to incorrect conclusions in some experiments. When extracellular Na^+^ is substituted by NMDG and Ca^2+^ is present as sole permeable cation at a concentration of 10 mM, ADPR induces Ca^2+^ influx but 2-APB does not. However, this is not due to an inhibition of Ca^2+^ permeation by 2-APB because isosmotic substitution of NMDG with sucrose restitutes Ca^2+^ currents (Kühn et al., 2017). Corresponding observations were made with 2-APB and the TRPV6 channel (Kovacs et al., 2012). Thus, it appears that NMDG blocks pore entry of 2-APB.

It is hoped that further comparison between *nv*TRPM2 and *h*TRPM2 and the study of pore chimeras will produce insight on the structural requirements that govern inactivation.

## Responses to H_2_O_2_ demonstrate the functional role of the NUDT9H domain

A key feature of all TRPM2 channel orthologs studied previously (which were all mammalian representatives without exception) is their activation in response to oxidative stress (Hara et al., 2002; Fonfria et al., 2004) that is experimentally simulated by the extracellular application of H_2_O_2_ (Wehage et al., 2002). Currently the most accepted hypothesis is that H_2_O_2_ activates the channel indirectly through an accumulation of intracellular ADPR (Perraud et al., 2005). This view is supported by inside-out patch-clamp experiments in which H_2_O_2_ apparently had no direct effects on human TRPM2 (Tóth and Csanády, 2010). In extension of this view, a recent study reported that H_2_O_2_ sensitizes *h*TRPM2 to the activation by physiological body temperatures; the sensitization is achieved by the oxidation of a methionine residue localized in the N-terminus of the channel (Kashio et al., 2012). This mechanism, under some experimental conditions and probably *in vivo*, may contribute to channel activation in response to oxidative challenges.

Since not only this specific methionine residue is also conserved in *nv*TRPM2 but also *nv*TRPM2 is more sensitive to ADPR than *h*TRPM2, it was expected to confirm H_2_O_2_ responses as well, and probably stronger and faster ones because accumulated ADPR should activate *nv*TRPM2 more easily than *h*TRPM2. The opposite findings were obtained. H_2_O_2_ completely failed to induce any currents. This could not be helped by increasing the concentration or the time of incubation of H_2_O_2_; *nv*TRPM2 presented itself as a channel highly sensitive to ADPR but entirely insensitive to H_2_O_2_ (Kühn et al., 2015).

Genetic manipulations of the NUDT9H domain in *h*TRPM2 have revealed that its function is easily disturbed by subtle changes. There are quite a few point mutations that render channels completely insensitive to ADPR. Several short sequences were deleted or substituted with the same result (Hara et al., 2002; Kühn and Lückhoff, 2004; Perraud et al., 2005). When analogous changes were introduced in *nv*TRPM2, again surprising findings were obtained. In no case, any change in the response to ADPR could be demonstrated. However, these manipulations produced channels that were now readily activated by H_2_O_2_. It is not worthwhile to summarize here the specific alterations of NUDT9H that were studied because it turned out that none of them contributes to the understanding of *nv*TRPM2 channel function. Instead, they gave rise to a radically different perspective on the role that NUDT9H plays in *nv*TRPM2, additionally guided by elegant experiments from Perraud et al. (2005) who co-expressed TRPM2 channels along with a cytosolic variant of the ADPR-degrading human NUDT9 enzyme. This co-expression suppressed the H_2_O_2_-induced activation of human TRPM2 which is accomplished by intracellularly accumulating ADPR. Therefore, we speculated that the NUDT9H domain of *nv*TRPM2 did not mediate the activation by ADPR at all; instead, it prevented the activation by H_2_O_2_ by degrading ADPR in the vicinity of the channel pore. This latter role would fit very well to the two critical residues (EF instead of IL) in the enzymatic domain; furthermore, all changes that created H_2_O_2_ sensitivity could then be interpreted as loss of ADPR degradation.

As a definite experimental test of the hypothesis, a *nv*TRPM2 channel variant was constructed in which the entire NUDT9H domain had been deleted (*nv*TRPM2-ΔNUD). The absence of large parts of the C-terminus may lead to unpredictable structural changes of the protein, possibly resulting in misfolding and aberrant surface expression. Accordingly, it was mandatory to verify the correct surface expression of this variant. It is later discussed that incidentally, these expression studies revealed unforeseen insight into the function of the human NUDT9H domain. Not unforeseen, however, but rather hoped for as confirmation of the tested hypothesis, were the results on *nv*TRPM2-ΔNUD. To begin with, the surface expression was almost normal which is prerequisite for further functional studies. These studies then revealed that sizeable currents were induced by ADPR, such that the absence of the NUDT9H domain did by no means preclude channel activation by ADPR. In further confirmation of the hypothesis, H_2_O_2_ proved as an effective current activator on *nv*TRPM2-ΔNUD, in line with a missing ADPR degradation in the absence of a NUDT9H domain. As a more direct proof for the catalytic activity of the NUDT9H domain of *nv*TRPM2 and for its role in preventing channel activation by H_2_O_2_, calcium imaging experiments were performed on HEK-293 cells in which *nv*TRPM2-ΔNUD was co-expressed together with one of the following NUDT9 variants (see Figure 1): an essential part of the human NUDT9 enzyme (aa 77–350), the isolated NUDT9H domain of *nv*TRPM2 (aa 1,289–1,551), or the isolated NUDT9H domain of *h*TRPM2 (aa 1,253–1,503). Stimulation with H_2_O_2_ resulted in Ca^2+^ influx through *nv*TRPM2-ΔNUD when the enzymatic inactive NUDT9H domain of *h*TRPM2 was co-expressed. In contrast, co-expression of the human NUDT9 enzyme as well as of the NUDT9H domain of *nv*TRPM2 drastically suppressed the H_2_O_2_ responses of *nv*TRPM2-ΔNUD (Kühn et al., 2016). Therefore, the evidence is compelling that the ADPRase activity of the NUDT9H domain in *nv*TRPM2 is of decisive functional relevance, as is in the opposite way the loss of ADPRase activity in *h*TRPM2, which has already been demonstrated with the analogous experiments by Perraud et al. (2005).

Thus, the original approach of studying species variants, to elucidate the apparently unique NUDT9H-directed activation of TRPM2 by ADPR, led to the realization that there exist two completely different mechanisms for ADPR-dependent channel gating (Kühn et al., 2016), one present in mammals and one in cnidarians.

**Figure 3:**
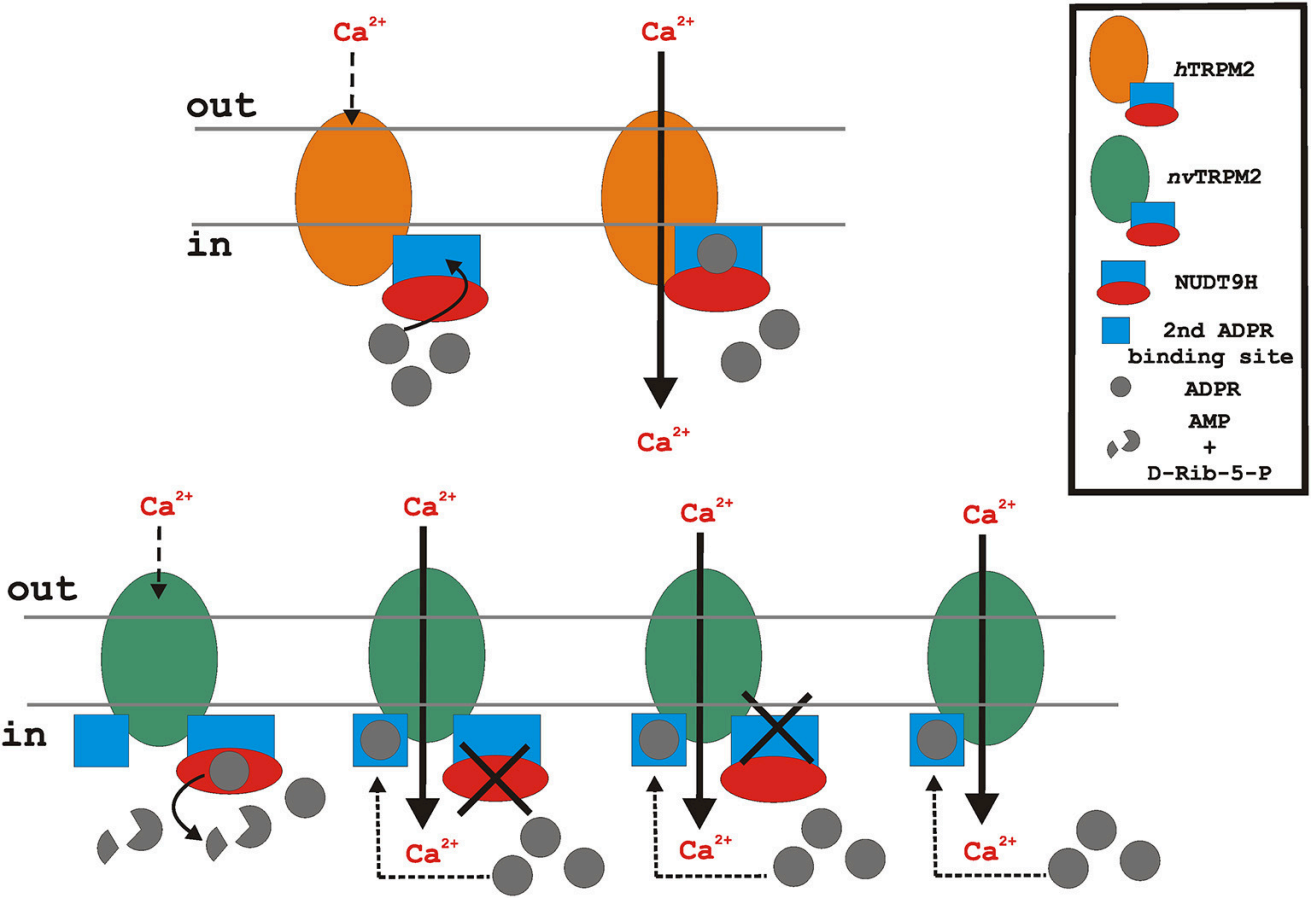
Putative functional role of the endogenous NUDT9H domain of the TRPM2 orthologs during oxidative stress (stimulation with H_2_O_2_). Oxidative stress leads to intracellular accumulation of ADPR. In the case of *h*TRPM2 (upper), ADPR binds to the cytosolic NUDT9H domain (lacking significant ADPRase activity) and initiates channel activation. In contrast, *nv*TRPM2 (lower, left) exhibits full catalytic activity. Therefore, ADPR is degraded and the cytosolic concentration of ADPR remains too low to initiate channel activation. However, when the enzymatic function of the NUDT9H domain of *nv*TRPM2 is disrupted due to point mutations, interfering either with binding or with cleavage of ADPR, or due to the deletion of the entire NUDT9H, the accumulated intracellular ADPR enables channel gating via a second interaction site. The cartoon reflects the experimental situation of *nv*TRPM2 over-expression in mammalian cells (HEK-293) with no external ADPR added to the cytosol. Note that for the proper function of *nv*TRPM2 in their native environment, a cellular mechanism is required that controls the catalytic activity of the NUDT9H domain and thereby enables ADPR-dependent gating of *nv*TRPM2.

The cartoon in Figure 3 is intended to illustrate the results on H_2_O_2_ stimulation of heterologously expressed wild-type *h*TRPM2, wild-type *nv*TRPM2, and of *nv*TRPM2 variants in which different parts of the NUDT9H domain have been modified or deleted.

Several questions immediately arise. The first one is how wild-type *nv*TRPM2 can show as extremely sensitive to ADPR in patch-clamp experiments (Kühn et al., 2015) when the NUDT9H domain degrades all ADPR in the vicinity of the channel. Possibly, its ADPRase activity is overpowered by the inexhaustible ADPR supply of the patch pipette. This interpretation is in line with the experimental findings of Perraud et al. (2005) where the ADPRase activity of the co-expressed human NUDT9 enzyme lost its relevance when increased concentrations of ADPR were used in the patch-clamp pipette.

As second question for which no easy answer is available at present, we have to ask how ADPR accomplishes gating in the absence of NUDT9H and whether there is an additional binding site for ADPR. It is plausible that such a binding site should be in the N-terminus as only longer intracellular region of *nv*TRPM2-ΔNUD.

## *nv*TRPM2—a prototype for a novel mechanisms of ADPR-directed channel activation

In principle, ADPR-dependent channel activation would not necessarily require a binding site. Alternatively, mechanisms like ADP-ribosylation should be discussed. PARPs, however, would be no good candidates which could achieve such a modification of an ion channel because they are all transferases and transfer the ADPR-moiety from the cofactor NAD^+^ to the protein (Barkauskaite et al., 2015); free ADPR is not a suitable substrate for PARP enzymes. Moreover, ADPR is able to induce channel gating in cell-free patches. Unless there would be membrane-associated enzymes that accomplished ADP-ribosylation of *nv*TRPM2, which seems somewhat remote in our opinion, the finding strongly contradicts such a mechanism (Kühn et al., 2016).

Potential binding sites might either exhibit known motifs for ADPR binding such as the Nudix box, or represent a new type of interaction. They are not expected to have ADPRase activity because the non-hydrolyzable ADPR analog AMPCPR is fully accepted as an activator (own unpublished results).

While Nudix box motifs cannot be found in the N-terminus or in other cytosolic parts of *nv*TRPM2, future search of binding sites may be guided by studies on a protein module ubiquitous in eukaryotes, bacteria, and archaea (Chakravarthy et al., 2005). The module was originally characterized in the histone variant macroH2A (Chakravarthy et al., 2005) and is well known for its capability to bind ADPR (Karras et al., 2005).

## Possible role of *nv*TRPM2 *in vivo*

Considering the unique functional properties demonstrated by the *nv*TRPM2 channel in HEK-293 cells, the question arises how a combination of an ADPR-sensitive channel and a catalytic active ADPRase function might work *in vivo* in the sea anemone. Concededly, it is not the primary goal of our present research or of this review to describe the physiological role that TRPM2 plays in *Nematostella vectensis*. Moreover, fundamental information is lacking that would put any speculation on such a role on a more solid basis. In particular, data on the spatial and temporal expression of TRPM2 in *Nematostella vectensis* are still missing. Nevertheless, some thoughts on this topic may be outlined here.

During evolution, the sea anemone has separated from man some 800 million years ago. Notwithstanding, there is a striking degree of conservation concerning the gene families in the genome of *Nematostella* and vertebrates, as revealed by expressed sequence tag (EST) and genome analyses. This indicates that many ancestral traits have been preserved in *Nematostella* (Genikhovich and Technau, 2009). For this very reason, *Nematostella vectensis* currently represents a model organism in which fundamental biological processes are intensely studied, such as axial patterning, plasticity of the nervous system or stress responses (Layden et al., 2016).

Certainly, oxidative stress plays an important role in sea anemones in their natural habitat (Goldstone, 2008; Reitzel et al., 2008; Tarrant et al., 2014). However, at present there is no information about the intracellular regulation of the ADPR concentration. In particular, it has not been proven that intracellular ADPR is mobilized by oxidative stress, as in mammalian cells, although PARPs and PARGs as well as the NUDT9 enzyme are represented in the genome of *Nematostella vectensis* (as derived from the Joint genome institute database). Thus, at least the signaling cascade that leads to the activation of a Ca^2+^-permeable, depolarizing cation channel as consequence to DNA damage, seems to be fully constituted in *Nematostella*.

**Figure 4:**
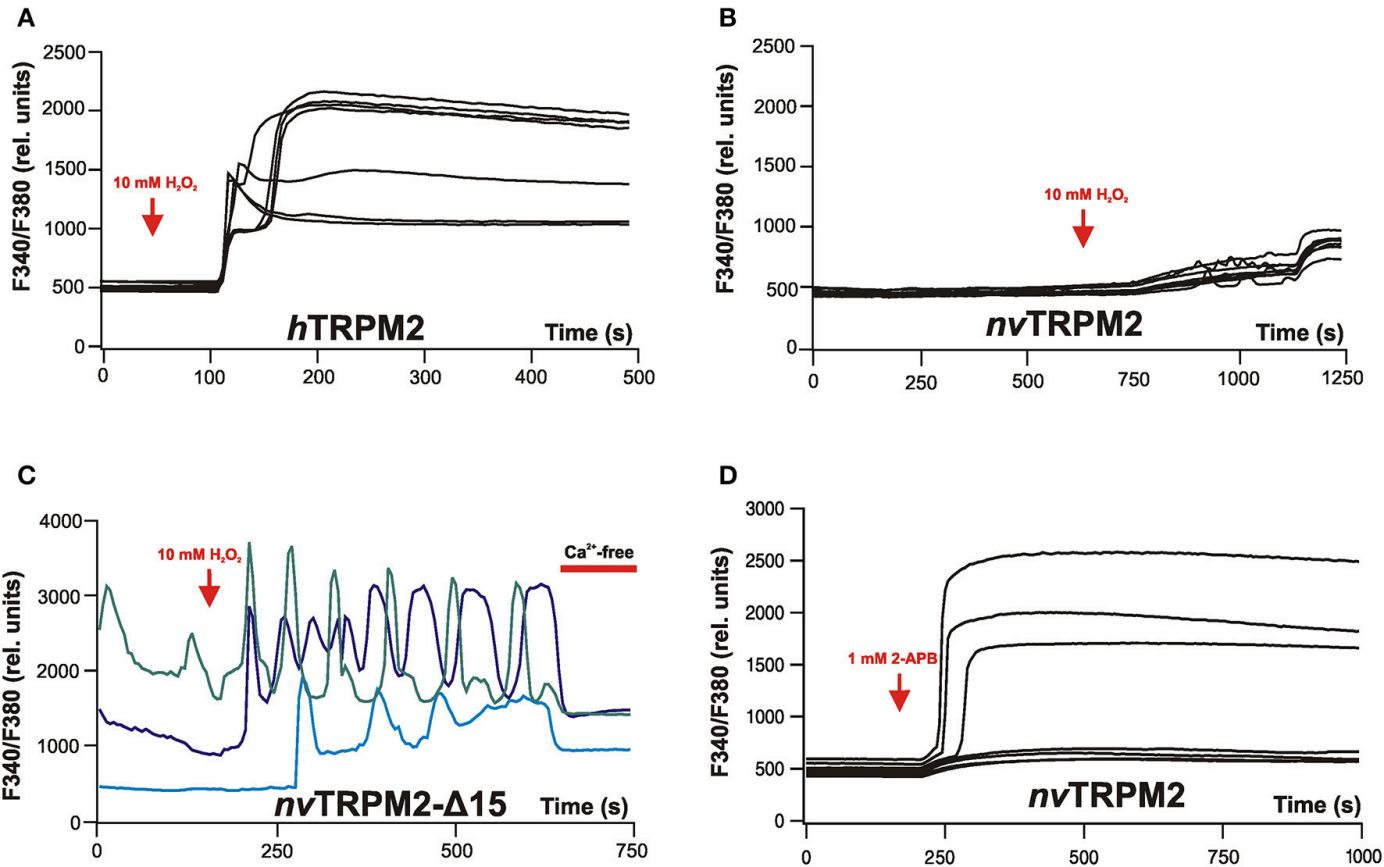
Calcium imaging reveals typical effects on intracellular Ca^2+^ concentrations evoked by stimulation of either *h*TRPM2 or of *nv*TRPM2 channels in HEK-293 cells. **(A)** Characteristic changes of the intracellular Ca^2+^ concentration of cells transfected with human TRPM2 after extracellular stimulation of the cells with 10 mM H_2_O_2_. Note the plateau-like increases in [Ca^2+^]_i_. **(B)** Cells transfected with *nv*TRPM2 do not respond to oxidative stress. **(C)** Cells are transfected with a channel variant of *nv*TRPM2 where the ADPRase activity of the NUDT9H domain has been disrupted by a deletion. H_2_O_2_ induces characteristic oscillations of [Ca^2+^]_i_. For better distinction, the individual curves are highlighted in different colors. After replacing the standard bath solution (containing 1.2 mM Ca^2+^) with a divalent-free bath solution (containing 10 mM EGTA) the oscillations stop. **(D)** Stimulation of *nv*TRPM2 by extracellular application of 2-APB (1 mM). A plateau-like increase in [Ca^2+^]_i_ results because 2-APB is an activator and prevents channel inactivation at the same time. Responses of non-transfected cells are shown as negative control. Figures are slightly modified from Kühn et al. (2015, 2017).

Speculations on how this cascade proceeds are nourished by the peculiar kinetics of *nv*TRPM2. In studies on human TRPM2, extracellular application of H_2_O_2_ as an experimental paradigm of oxidative stress leads to an extended channel activation, resulting invariably in a permanent and massive elevation of the intracellular Ca^2+^ concentration (Figure 4A). This kind of response fits well to other observations in which H_2_O_2_ induces apoptosis in a TRPM2-dependent manner (Miller and Zhang, 2011; Naziroglu, 2011; Takahashi et al., 2011). In contrast, the consequences of *nv*TRPM2 activation *in vivo* are anticipated to be far less drastic than cell death because channel activation is short and followed by immediate inactivation. This is already evident in patch-clamp experiments. An approach that is closer to a physiological situation are calcium imaging experiments because the cytosol is left intact and can be controlled and regulated by the cells. However, an ADPR-mediated stimulation of *nv*TRPM2 is not feasible in such experiments because ADPR cannot be applied intracellularly and because application of H_2_O_2_ is without effect on wild-type *nv*TRPM2 (Figure 4B). As discussed, this lack of *nv*TRPM2 response, which occurs in spite of the high ADPR sensitivity, is due to the degradation of ADPR by the catalytic active NUDT9H domain. When the enzymatic activity is abrogated by genetic manipulations, e.g., by deletion of the entire NUDT9H domain, H_2_O_2_ becomes effective and evokes increases in [Ca^2+^]_i_ by Ca^2+^ entry through the *nv*TRPM2 variants. As expected, these Ca^2+^ responses are characterized by a lag time, by a rapid increase of [Ca^2+^]_i_ after the lag time, and by a fast decline. Moreover, oscillations of [Ca^2+^]_i_ are consistently found that display as sharp peaks of [Ca^2+^]_i_, fast returns to baseline, and extended periods at baseline level prior to the next sharp peak (Figure 4C). In many experiments, such oscillations were observed already in the absence of H_2_O_2_. Therefore, the basal ADPR concentration in the chosen cell model for heterologous overexpression (HEK-293) is sufficient for *nv*TRPM2 stimulation, provided the ADPR degradation by the NUDT9H domain is prevented.

Without doubt, [Ca^2+^]_i_ oscillations play a pivotal role in many important physiological processes (e.g., circadian rhythm, fertilization) and oscillatory Ca^2+^ signaling associated with endogenously expressed TRPM channels has been described in *Caenorhabditis elegans* (Xing and Strange, 2010); hence, the finding may well be meaningful for the physiological role of TRPM2 in the sea anemone. Unfortunately, there are still a lot of fundamental questions about the oscillations.

Primarily, it is unclear how degradation of ADPR by the NUDT9H domain in *nv*TRPM2 should be prevented in *Nematostella in vivo*. One way how this might happen was demonstrated by Carloto et al. (2006) in studies with the human NUDT9 enzyme. In the presence of H_2_O_2_, the preferred divalent cation for the ADPRase activity becomes Mn^2+^ rather than Mg^2+^ which then can no longer act as cofactor; the result is an increased *K*_m_ for ADPR. Accordingly, treatment with H_2_O_2_ virtually abolishes the enzymatic activity with Mg^2+^ as cofactor (Carloto et al., 2006).

Mechanistically, it is not easy to understand how the [Ca^2+^]_i_ oscillations are accomplished. Channel inactivation is certainly a key element because 2-APB (1 mM) induces no oscillations but instead causes a permanent increase in [Ca^2+^]_i_ (Figure 4D). However, patch-clamp experiments have so far not revealed how inactivation can be temporarily reversed, which seems to be a prerequisite for oscillations. Moreover, this must happen in an extremely homogenous and synchronized manner within the total channel population of a cell. Cyclic regulation of the ADPRase activity of the NUDT9H domain cannot be an explanation because oscillations were observed exclusively in mutants where this region is dysfunctional. Ca^2+^-dependent regulation of other ADPR-degrading enzymes is a theoretical possibility without experimental evidence.

So there are ample research opportunities for scientists fascinated by the biology of the sea anemone and by the signaling that *nv*TRPM2 may participate in. Our own interests are focused more on what is outlined in the following chapter.

## Lessons to be learned from *nv*TRPM2 for the gating mechanism of *h*TRPM2

When we started the studies on *nv*TRPM2, our long-lasting and general aim was gain of insight on the relation between structure and function of the human TRPM2 channel, with emphasis on gating as consequence of ADPR binding. We thought we were on a promising path when we achieved the functional expression of *nv*TRPM2 as ADPR-activated channel. Then we discovered that the NUDT9 homology region fulfills opposite functions in sea anemone and man, dampening the hope for learning with this approach how the C-terminus contributes to gating.

Obviously, evolution had used strongly divergent paths to create an ADPR-gated channel in cnidarians and mammals which could not be anticipated. In spite of the unexpectedly large functional inter-species discrepancies, our research on *nv*TRPM2 directed us toward experiments on the human C-terminus that reveal valuable information on the function of this domain so different from *Nematostella*.

Although many structural requirements for ADPR binding have been defined in *h*TRPM2 as well as in the NUDT9 enzyme, the most urgent question remains how ADPR binding to the channel creates the structural re-arrangement decisive for gating and which parts of the protein participate.

In a recent study, a detailed structural model for the binding of ADPR to the NUDT9H domain of the *h*TRPM2 channel was proposed (Yu et al., 2017). This model is basically guided by the crystal structure of the human NUDT9 enzyme (Shen et al., 2003). However, as promising as this approach may be, a structural view on the isolated NUDT9H domain entails the risk of misinterpreting the situation in the full-length channel. This reservation does not concern a potential design of drugs that might modify the function of the NUDT9H domain. But this approach does not necessarily elucidate the structural basis of the interaction between the NUDT9H domain and the other parts of the channel. Hints that may shed light on this interaction derive from our experimental findings that certain C-terminal modifications of *h*TRPM2 interfere with channel function (Kühn et al., 2015, 2016). These findings demonstrate that not only subtle alterations within the NUDT9H region have a strong impact on the human channel but also manipulations of the C-terminus outside of this region. Importantly, these modifications appear to compromise the NUDT9H domain independently of the function that the region fulfills in each species. In *h*TRPM2, gating is prevented, whereas ADPR degradation is abolished in *nv*TRPM2.

In future, it will be a major challenge to integrate all the experimentally gained information on particular mutated single amino acids in *h*TRPM2 and *nv*TRPM2 into structural models that may help to explain the interaction of various parts of the whole protein on a mechanistic level.

With respect to the yet unknown interaction mode between NUDT9H and channel core in *h*TRPM2, the disturbance of the NUDT9H domain by modifications outside of it raises further questions. It should be studied in detail whether binding of ADPR is impeded or whether a subsequent step within the gating process is affected. Iordanov et al. (2016) have already presented evidence that within the NUDT9H region, about 20% of C-terminal sequence might represent an interface for the transduction of ligand binding to pore-opening. It is imagined that larger parts of the protein participate geometrically to orchestrate a fully functional interaction.

In general, the comparison of *nv*TRPM2 and *h*TRPM2 remains an attractive approach to delineate the structural basis for particular functional details, but these studies are only at their beginning.

## Concluding remarks

In the sea anemone *Nematostella vectensis* as well as in mammals, TRPM2 represents a cation channel activated by ADPR. This mode of channel activation is unique and not found for any other known channels. However, the mechanisms how ADPR achieves gating are remarkably distinct in the orthologs, and opposite tasks have been assigned to the NUDT9H domain. Hence, TRPM2 is a fascinating example how one gene in distantly related species has evolved in a strikingly divergent manner and still has gained analogous functional properties. At the same time, evolution has created critical but diametrically different roles for homologous parts of the protein. In the NUDT9 domain of *nv*TPM2, as opposed to the situation in *h*TRPM2, catalytic function is conserved and bears functional importance for channel function. Thus, *nv*TRPM2 can be considered a true and unquestionable chanzyme.

## Author contributions

All authors listed have made a substantial, direct and intellectual contribution to the work, and approved it for publication.

### Conflict of interest statement

The authors declare that the research was conducted in the absence of any commercial or financial relationships that could be construed as a potential conflict of interest.
